# Supplementary material for: Maize Internode Autofluorescence at the Macroscopic Scale: Image Representation and Principal Component Analysis of a Series of Large Multispectral Images
Source: Biomolecules. 2023 Jul 11;13(7):1104. doi: 10.3390/biom13071104 (PMC10377703; doi:10.3390/biom13071104)
Supplement: Supplementary file 1 [file biomolecules-13-01104-s001.zip › Supplementary3-score1.pdf]

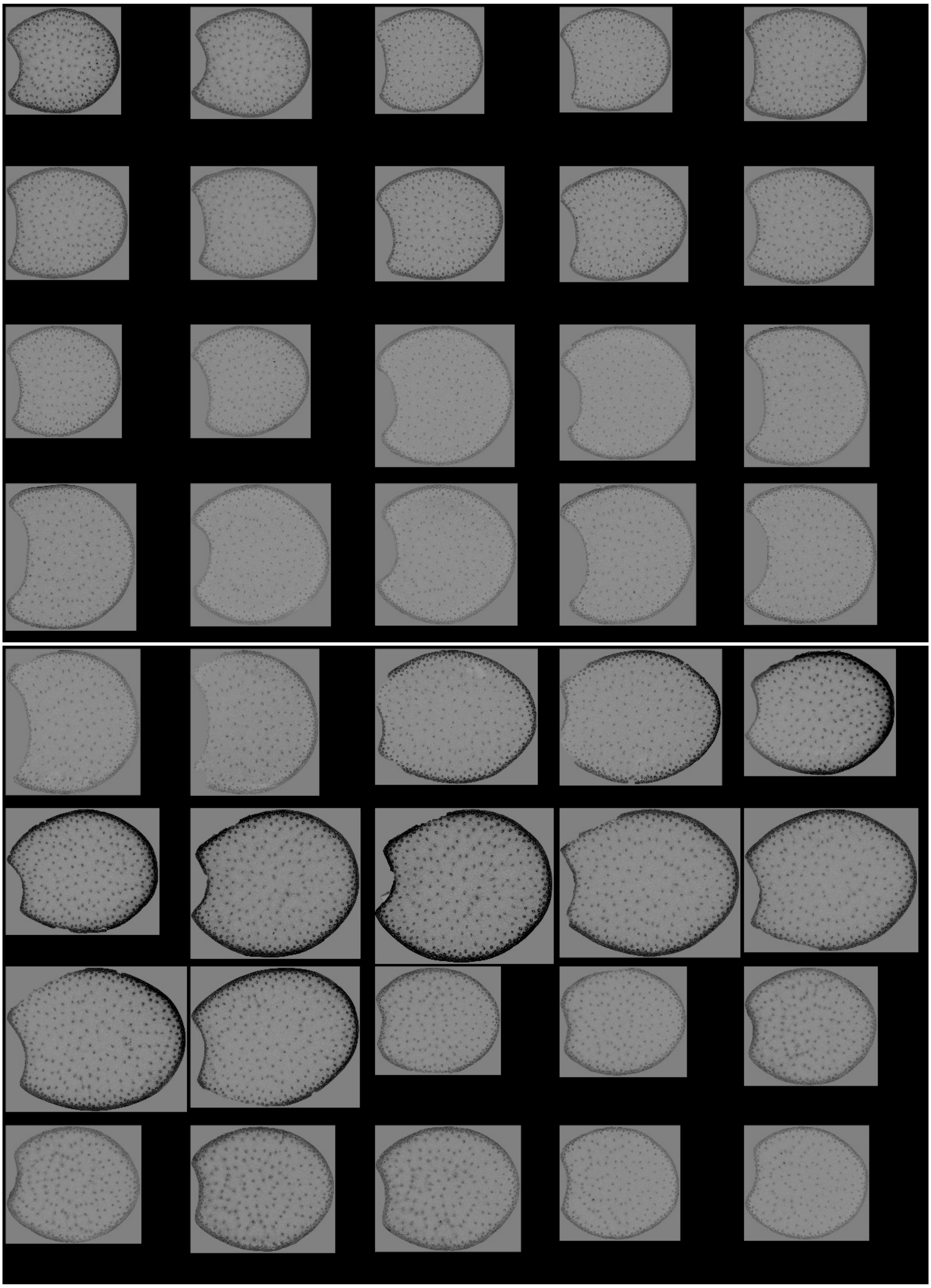

Large PCA. Principal Component 1. Montages of the 40 score images of the series. Field of view of each image montage: 7.89 cm x 11.45 cm corresponding roughly to 2 x 2 cm² per section. Intensities can be compared.

Montage 1: images 1 to 12: inbred line M21 with 2, 2, 3, 3 and 2 sections per internode, respectively. Images 13 to 20: inbred line M22 with 2 sections per internode.

Montage 2: images 1 to 2: inbred line M22 with 2 sections per internode. images 3 to 12: inbred line M23 with 2 sections per internode. Images 13 to 20: inbred line M24 with 2 sections per internode. Only 4 internodes could be analysed for this line

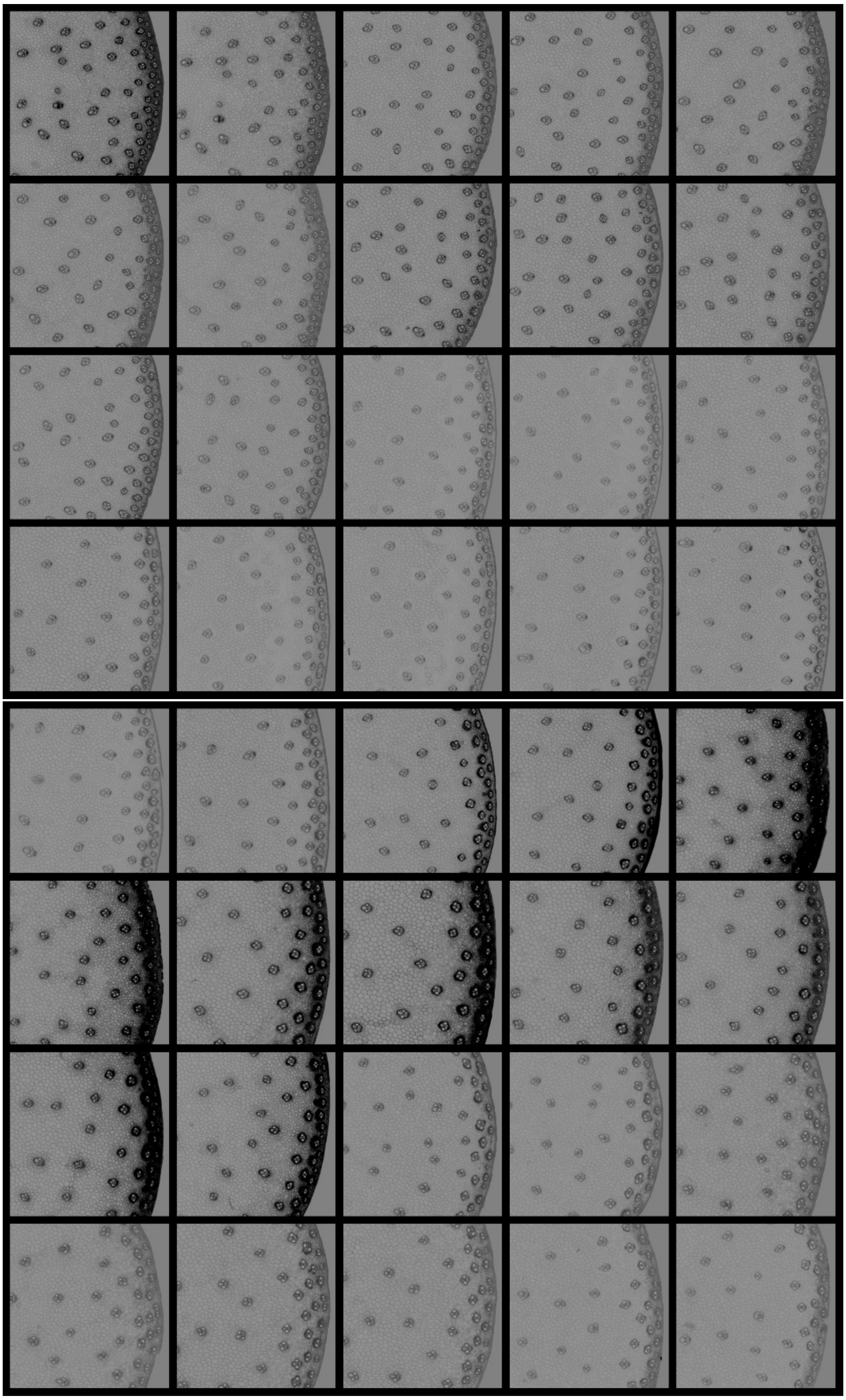

Large PCA. Principal Component 1. Montages of the 40 rind zoomed score images of the series. Fields of view: one image: 4.17*4.17 mm², montage: 1.89 cm x 2.28 cm. Intensities can be compared.

Montage 1: images 1 to 12: inbred line M21 with 2, 2, 3, 3 and 2 sections per internode, respectively. Images 13 to 20: inbred line M22 with 2 sections per internode.

Montage 2: images 1 to 2: inbred line M22 with 2 sections per internode. images 3 to 12: inbred line M23 with 2 sections per internode. Images 13 to 20: inbred line M24 with 2 sections per internode. Only 4 internodes could be analysed for this line

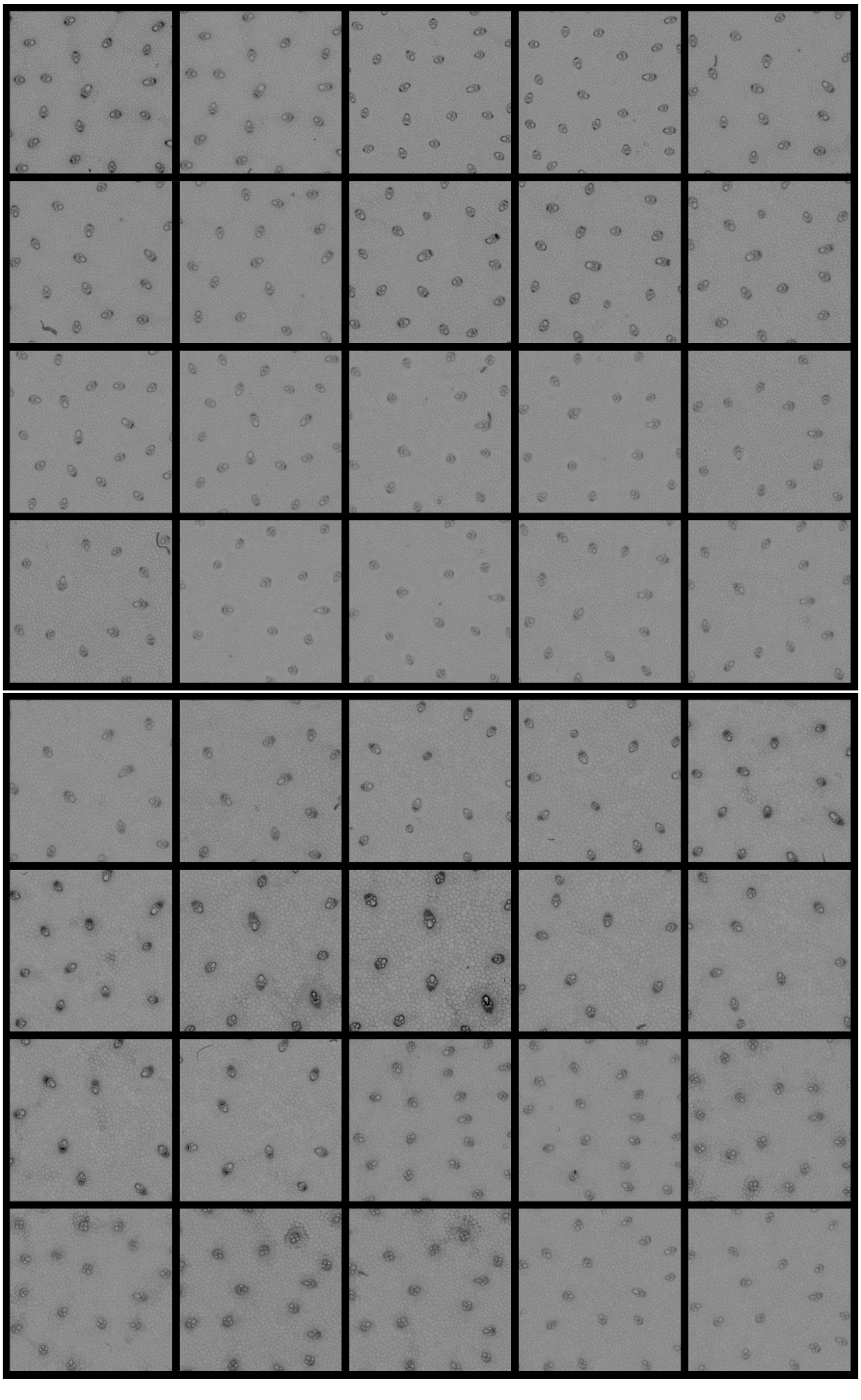

Large PCA. Principal Component 1. Montages of the 40 centre zoomed score images of the series. Fields of view: one image: 4.17*4.17 mm², montage: 1.89 cm x 2.28 cm. Intensities can be compared.

Montage 1: images 1 to 12: inbred line M21 with 2, 2, 3, 3 and 2 sections per internode, respectively. Images 13 to 20: inbred line M22 with 2 sections per internode.

Montage 2: images 1 to 2: inbred line M22 with 2 sections per internode. images 3 to 12: inbred line M23 with 2 sections per internode. Images 13 to 20: inbred line M24 with 2 sections per internode. Only 4 internodes could be analysed for this line
